# The fight against COVID-19: a report from the Italian trenches

**DOI:** 10.1017/S1041610220000630

**Published:** 2020-04-20

**Authors:** Diego De Leo, Marco Trabucchi

**Affiliations:** 1 Italian Psychogeriatric Association, Brescia, Italy; 2 De Leo Fund, Padua, Italy; 3 Australian Institute for Suicide Research and Prevention, Griffith University, Mount Gravatt, Australia; 4 Tor Vergata University, Rome, Italy

Our senior citizens are the most exposed to the consequences of the COVID-19. Frailty caused by comorbidities makes the advanced age of many people particularly vulnerable to the infection of this new coronavirus, the characteristics of which are still largely unknown to fellow scholars. What is clear is that older adults die more frequently than younger age groups, everywhere in the world. In the Veneto region, 36.3% of individuals who died were 85+ years old, with the mean age of those who lost their lives to coronavirus being 81 years (Regione del Veneto, 2020). In the last days, Italian citizens were hugely impressed by watching on television a long series of military trucks transporting the coffins of dead people away from their home places because there is no more room in the close cemeteries or opportunities for cremation. The mental health of our seniors is particularly challenged: they feel scared by the news and aware that, if infected, they would not receive the same attention (e.g. intubation, a bed in resuscitation unit, etc.) of younger individuals. Older adults with mental health conditions feel more frail and vulnerable than before: contacts with carers are now reduced to the minimum, with loneliness and abandonment becoming an excruciating reality. Checking on regular assumption of drug therapies may become problematic; eating properly and keeping with personal hygiene at a sufficient level can also be quite difficult. This may increase the sense of demoralization and despair in people. A few cases of suicide have been signaled by media (Gazzettino, 2020), but in the present chaotic situation, it would not be surprising if similar fatalities would remain mostly undetected.

Demented people are particularly exposed to the impact of COVID-19; there are anecdotal reports from domiciliary care nurses and staff in nursing homes that cases of delirium are on sharp increase. This could be justified by the positioning of the virus in the central nervous system (Li *et al.*, 2020). On the other hand, in the present situation, caregivers of people with dementia are also exposed to extra stress: limited opportunities to offer the usual level of care; food and cleaning management more problematic; worries and concerns for the possibility of contaminating an older adult that would not survive the disease; and, in a situation like the Italian one, the many “badanti” (carers from Eastern Europe) without a regular contract (Rugolotto *et al*., 2017), now impeded to reach the home of the older adults they take care of because they are intercepted by the police at check points.

## A world hecatomb

While we are writing this commentary from Italy (April 10th), it appears that the number of people tested positive to the virus in the United States has almost quadrupled the number of people found positive in our country; however, Italy counts a disproportionately high number of deaths. China – the country where everything reportedly originated – has now smaller figures than those of the US, Italy, Spain, and Germany. These countries, together, make more than one million people detected as infected (Worldometers, 2020). Not much can be said about the reliability of these figures or their comparability. It is good to say that the numbers are based on subjects who tested positive to the swab. Unfortunately, Italy has lost the opportunity to keep reliable records of those subjects. So, it is not known what is the percentage of those who repeated the test several times but are represented as different individuals; equally unknown, because not standardized, are the criteria for which the tests were performed, if to patients with initial symptoms (Fever? Fever and coughing? Fever, coughing, and dyspnea? What?); with “full-blown” clinical syndromes (severe symptomatology); to individuals potentially exposed by contiguity or direct contact (living in the same environment does not necessarily imply proximity or contact), etc. In essence, it is not known in which of these situations the swabs have been made. In this way – we believe – a big opportunity to study contagion modalities has been lost. Each region of Italy has run autonomous strategies to protect its citizens and counteract the disease. This has applied also to test-related policies. Thus, aggregating data from regions looks like a problematic, though unreliable, process.

We were expecting tests to be performed to all health personnel of common health settings: from general medicine surgeries to protected residences (including those for older adults), to emergency departments, and to general hospitals. Unfortunately, this elementary strategy to guarantee that health professionals were sufficiently protected toward the virus was not applied with due care, and up to date the number of doctors who died from the infection during their professional activity is unbearable (109 victims, at the time of writing). The number of other health workers who have lost their life is also tragically unbearable: 28. It has been reported that the costs involved in performing the tests in appropriate numbers (at least to all health professionals and people presenting with light symptoms) are too heavy, and the availability of skilled technicians and reagents for carrying out the tests is scarce.

## Lack of preparedness to the pandemic

In Italy, and probably elsewhere, nothing is and will be like before the pandemic. For the moment, we must overcome the crisis, but there could be a lot to say, on a clinical level, on that of the organization of services, and on how to protect the life of our older adults. The latter have been too affected by imprudent and superficial colleagues to feel accepted by the community again; in a few days, in deciding if the death was “by coronavirus” or “with coronavirus,” we have undone decades of geriatric education, but above all, we have destroyed a fundamental relationship of trust. How will we approach patients if they are now convinced that today we ask for their age to make critical decisions (intubation) and maybe tomorrow the administration of expensive drugs? A discrepancy between the gift of a long life and public acceptance of older adults is increasingly noticeable and clearly expressed by the deprecated document on “damages caused by longevity,” which Christine Lagarde (now the president of the European Central Bank) spoke about a few years ago (2012): “Old people live too long and this is a risk for the global economy. We must do something, urgently.”

The scenario we are witnessing today is characterized by different situations in which people affected by COVID-19 are forced to live. The main ones are schematically summarized below, even if there are significant differences, related to the different organization of services in the regions:

A. Older adults who remain at home even when the first symptoms appear are entrusted in most cases to themselves and to the care of their loved ones. Family doctors are afraid; they tend to block visits, and families do not call emergency services because they fear seeing the family member get on the ambulance and not being able to greet him/her before they die. So they keep the sick – especially if a senior – in their homes, assist them with fear and affection until the negative or positive resolution of the clinical picture. There is no mention of swabs or blood chemistry analyses, let alone CT or other scans. Our fellow citizens risk ending their lives without even being told what is happening to them. Many seniors live alone; many have limited or no familiarity with the Internet and are poorly connected with other family or community members (Newman and Zainal, 2020). Some die in complete isolation and their corpses are discovered many days after death (Gazzettino, 2020).

B. In the last couple of months, the people who live in nursing homes have been completely forgotten by administrative powers. Frail older adults are certainly the subjects who have suffered the most from the difficulties of the Italian health system. In cities of Lombardy such as Bergamo and Brescia, the deaths have followed at an impressive rate giving rise to sad images in the televisions of the Italians of long lines of military trucks carrying coffins to incinerators, often very far from the place of origin of the deceased. As reported elsewhere (Trabucchi and De Leo, in press), in just 20 days, in the nursing homes of the province of Bergamo (Lombardy), which have a total accommodation capacity of 6,400 beds, there were more than 600 deaths, which is a hecatomb. The real dimensions of the phenomenon will only be known after some time; it is probable that the number of infected people is far greater than that officially reported. It is also likely that the number of deaths is far greater than that officially attributed to COVID-19. No attention was paid to those who manage the residences, no support in terms of means of protection, the possibility of making swabs, or even economic support in the face of damage caused by the release of beds caused by the death of guests. The access of new guests has been blocked, but the residences are increasingly pressed to admit elderly people discharged from hospitals. The staff is exhausted, as a consequence of heroic commitment. In small communities, the warmth that stirs around the residences compensates for the solitude of guests and operators, but this is not the case in large centers, where the bigger turnover of personnel makes the atmosphere more impersonal. In any case, since doctors and other operators get sick in rapid progression – and despite the extreme commitment of those on duty – guests feel progressively more fearful and abandoned.

C. In Italy, hospitals are at the center of the COVID-19 cyclone: the attention of the media and the whole nation is focused on them. Schematically, the hospital system moves according to this model: (a) the emergency department, where patients arrive after days spent at home, sometimes already exhausted by the disease. It is rarely possible to send them back home: in most cases, they are kept under observation, waiting for a bed; (b) resuscitation units, where patients are intubated and followed mainly by doctors specialized in anesthesia and resuscitation; and (c) departments where patients are treated with oxygen therapy (with different levels of intensity) and checked for overall health conditions, including psychological problems. At this point in time, all remaining wards for the management of the diseases that “normally” refer to hospitals are in progressive reduction. To cope with the emergency, resuscitation units were built in record time, and entire hospitals were reconverted into the care of COVID-19. Unfortunately, the pandemic has found the country largely unprepared and unable to provide the necessary protection in time even to its health workers, resulting in serious shortcomings regarding the supply of eyeglasses, masks, gloves, and gowns. As said, this has resulted in the loss of far too many health professionals’ lives. The very serious difficulties in assisting critical situations, combined with the scarcity of places suitable for the reception of patients in severe conditions and the lack of a sufficient number of ventilators, have given rise to very painful ethical choices for health professionals on whom to privilege in the care with available equipment (Rosenbaum, 2020).

D. From hospitals, patients are discharged when they achieve clinical recovery; they can return home, or to low-intensity “hotels,” where they can manage themselves in isolation. The problem of older adults remains unsolved; often, after reaching clinical recovery, they show such a degree of disability that returning home is not possible, and it is difficult to resort to rehabilitation or long-term care facilities.

## Looking ahead

The described picture seems to apply to many northern territories of Italy. Any criticism must be postponed; however, we seriously doubt that transferring people discharged from hospitals to rest homes may represent an appropriate choice. And certainly we should find a way to respond to those families that cannot accompany their loved ones to Intensive Care Units and are left without any information and prognostic indication. This often means that the patients were kept too long at home, without effective tools to catch the signs of aggravation in time, which is frequently sudden and characterized by the discrepancy between symptoms and objectivity (e.g. oxygen saturation or temperature).

The south of Italy is generally less equipped than the north of the country, and grave concerns rest on a possible dramatic evolution of the next few days over there. Social distancing and isolation remain at the moment the only strategies available to citizens. However, the latter need to feel supported by governments; mental health should soon regain a very high status on the agenda of all nations, especially if confinement at home expects to be very long, and financial, family, and relationship problems risk to aggravate a future now seen with serious concerns and deep anxieties.

There are too many unanswered questions regarding the disease, and even the duration and quality of immunity post-infection remain a big question mark. While waiting for a vaccine to be ready, several protocols and drugs are being proposed by clinicians. None – at the moment – appears as particularly promising. However, we remain confident that a serious and open collaboration among researchers, institutions, and countries across the globe will offer concrete hope for our future.

For the time being, the mental health of people needs to be supported in any possible way. Social distancing, loneliness, forced isolation, and fear of contracting the illness are all big challenges for the general population facing the expansion of the epidemic, but the risk of psychological consequences can be greater for the frail senior (Armitage and Nellums, 2020). We need to activate all possible opportunities to offer help, at least in the form of tele-assistance, to our patients (Krysinska and De Leo, 2007). Psychological support should be made available to all via NGOs and public services, with contact with psychiatrists and other physicians actively established. As much as possible, health professionals should contact their patients and make the continuity of care a reality as soon as possible. Active outreach seems to be imperative, especially for older adults (De Leo *et al*., 2002), in order to counteract feelings of abandonment and disempowerment that COVID-19 is imposing on all community members, especially to the most fragile ones.
